# Converging evidence points towards a role of insulin signaling in regulating compulsive behavior

**DOI:** 10.1038/s41398-019-0559-6

**Published:** 2019-09-12

**Authors:** Ilse I. G. M. van de Vondervoort, Houshang Amiri, Muriel M. K. Bruchhage, Charlotte A. Oomen, Nitin Rustogi, Jason D. Cooper, Jack J. A. van Asten, Arend Heerschap, Sabine Bahn, Steven C. R. Williams, Jan K. Buitelaar, Geert Poelmans, Jeffrey C. Glennon

**Affiliations:** 1 Department of Cognitive Neuroscience, Donders Institute for Brain, Cognition and Behaviour, Radboud University Medical Center, Nijmegen, The Netherlands; 2 Neuroscience Research Center, Institute of Neuropharmacology, Kerman University of Medical Sciences, Kerman, Iran; 3 Department of Radiology and Nuclear Medicine, Radboud University Medical Center, Nijmegen, The Netherlands; 4 Centre for Neuroimaging Sciences, Institute of Psychiatry, Psychology and Neuroscience, King’s College London, London, UK; 5 Centre for Neuroscience, Swammerdam Institute for Life Sciences, University of Amsterdam, Amsterdam, The Netherlands; 6 Department of Molecular and Cellular Neurobiology, Center for Neurogenomics and Cognitive Research, VU University Amsterdam, Amsterdam, The Netherlands; 7 Cambridge Centre for Neuropsychiatric Research, Department of Chemical Engineering and Biotechnology, University of Cambridge, Cambridge, UK; 8 MRC Centre for Neurodevelopmental Disorders, King’s College London, London, UK; 9 Karakter Child and Adolescent Psychiatry University Centre, Nijmegen, The Netherlands; 10 Department of Human Genetics, Radboud University Medical Center, Nijmegen, The Netherlands; 11 Department of Molecular Animal Physiology, Donders Institute for Brain, Cognition and Behaviour, Radboud Institute for Molecular Life Sciences (RIMLS), Radboud University, Nijmegen, The Netherlands

**Keywords:** Molecular neuroscience, Psychology, Psychiatric disorders

## Abstract

Obsessive–compulsive disorder (OCD) is a neuropsychiatric disorder with childhood onset, and is characterized by intrusive thoughts and fears (obsessions) that lead to repetitive behaviors (compulsions). Previously, we identified insulin signaling being associated with OCD and here, we aim to further investigate this link in vivo. We studied TALLYHO/JngJ (TH) mice, a model of type 2 diabetes mellitus, to (1) assess compulsive and anxious behaviors, (2) determine neuro-metabolite levels by 1 H magnetic resonance spectroscopy (MRS) and brain structural connectivity by diffusion tensor imaging (DTI), and (3) investigate plasma and brain protein levels for molecules previously associated with OCD (insulin, Igf1, Kcnq1, and Bdnf) in these subjects. TH mice showed increased compulsivity-like behavior (reduced spontaneous alternation in the Y-maze) and more anxiety (less time spent in the open arms of the elevated plus maze). In parallel, their brains differed in the white matter microstructure measures fractional anisotropy (FA) and mean diffusivity (MD) in the midline corpus callosum (increased FA and decreased MD), in myelinated fibers of the dorsomedial striatum (decreased FA and MD), and superior cerebellar peduncles (decreased FA and MD). MRS revealed increased glucose levels in the dorsomedial striatum and increased glutathione levels in the anterior cingulate cortex in the TH mice relative to their controls. Igf1 expression was reduced in the cerebellum of TH mice but increased in the plasma. In conclusion, our data indicates a role of (abnormal) insulin signaling in compulsivity-like behavior.

## Introduction

Obsessive–compulsive disorder (OCD) is an often-debilitating condition, characterized by obsessive and/or compulsive behaviors. Obsessive behaviors include recurrent, intrusive, persistent thoughts, impulses and/or ideas that often cause anxiety or distress, and compulsive behaviors are ritualized, stereotypic behaviors, or mental acts performed to relieve anxiety or distress associated with the obsessions or according to rigid rules^1^. Although its exact etiology is still unknown, both genetic^2^ and environmental^3^ factors can contribute to OCD. The neuronal basis of the disorder is argued to be an imbalance in the activity of the cortico–striato–thalamo–cortical loop, resulting in hyperactivation of the orbitofrontal–subcortical pathway^4^. Imaging studies indeed showed increased activity (both in resting state and evoked) in the lateral and medial orbitofrontal cortex (OFC)^5,6^, the head of the caudate nuclei^5,7–9^ and the anterior cingulate cortex (ACC)^10–13^ in OCD patients. In addition, the cerebellum is emerging as a brain region of interest for OCD^14–16^. Furthermore, disturbances in the prefrontal cortex networks have been reported to contribute to disrupted cortical–striatal–cerebellar circuits in OCD^17^.

Recently, we have reported that altered central nervous system (CNS) insulin signaling may play a role in OCD^18^. We built a molecular landscape based on genome-wide association studies of OCD^19,20^. In this landscape, we identified insulin-related signaling and its downstream PI3K/AKT/RAC1 cascades as key players in OCD etiology, eventually affecting dendritic spine and synapse formation^21^. This finding is supported by the notion that OCD symptoms are associated with dysregulated peripheral insulin signaling, i.e. diabetes mellitus. Increased obsessive symptoms were observed in patients with type 1 diabetes mellitus (DM1)^22^ and OCD symptoms and glycosylated hemoglobin levels—a measure of type 2 diabetes mellitus (DM2) severity— showed a positive correlation^23^. The most important role of peripheral insulin is regulating blood glucose concentrations, but glucose uptake in the CNS is largely independent of insulin, and this is because the glucose transporters in the blood brain barrier are not regulated by insulin^24,25^.

That being said, insulin has important, non-metabolic functions in the CNS, including modulating neuronal survival^26^, synaptic and dendritic plasticity^27–29^, learning and memory^30,31^, and neuronal circuitry formation^32^. These functions are executed through a number of cascades, including the PI3K/AKT and RAS/MAPK pathways^21,33^. There are two sources of insulin in the brain: (1) it can be synthesized in the pancreas and enter from the periphery by crossing the BBB^34^, and (2) it can be synthesized in the CNS locally^35^. Moreover, there is a growing body of evidence pointing toward insulin-related signaling having an effect on white matter microstructure in the brain, which is assessed by diffusion tensor imaging (DTI). For example, insulin resistance in generally healthy middle-aged and older adults is associated with white matter microstructural alterations^36^ In young adults, hyperglycemia is also associated with increased white matter hyperintensities^37^. In addition, many studies report an association between DM1 or DM2 and white matter microstructure changes^38,39^.

In this study, we aimed to elucidate the link between DM2 and compulsivity-like behavior in the TALLYHO/JngJ (TH) mouse model at the behavioral, anatomical, metabolic, and molecular levels. TH mice mimic human DM2, as they develop hyperglycemia, hyperinsulinemia, and enlargement of the islets of Langerhans^40,41^. We combined behavioral tests, DTI, magnetic resonance spectroscopy (MRS), and proteomic assays of specific brain regions in the cortical–striatal–cerebellar loop, and found that disturbed insulin signaling contributes to the development of compulsivity-like behavior.

## Materials and methods

### Mice

TH mice and SWR/J mice, the control strain due to sharing the highest level of genetic homogeneity with TH mice^42^, were obtained from The Jackson Laboratory (Bar Harbor, ME, USA) and experiments started at 13 weeks of age (*n* = 9 per strain, male). The animals were individually housed (Blueline IVC without top, Tecniplast, Buguggiate, Italy) with crushed corncob bedding (The Andersons, Maumee, OH, USA), sizzle nesting material (Datesand Ltd., Manchester, UK), and an amber mouse igloo shelter (Datesand Ltd.). Housing was on a reversed day–night cycle (lights on at 20.00 h) in a ventilated cabinet with a light adjustment kit (Scantainer, Scanbur, Karlslunde, Denmark) and ad libitum food (V1244-703, SSNIFF spezialdiäten GmbH, Soest, Germany) and autoclaved demineralized water. Experimental procedures were conducted after approval of the Animal Ethical Committee of the Radboud University, Nijmegen, The Netherlands (project DEC2014-113), an approval in which the required sample size to ensure adequate power was estimated. A random number generator was utilized to randomize the location of the home cages in the cabinet and testing order during the experimental procedures.

### Behavioral studies

After one week of acclimatization to their new environment and being handled, the mice were used for the experiments conducted in a room solely lit by an infrared lamp (Y-maze and marble burying) or in a brightly lit room (open field and elevated plus maze). After randomization, the animals were tested in the same order during all experiments. The experiments were videotaped using a camera mounted above the maze and Media Recorder software (Noldus, Wageningen, The Netherlands). The activity patterns of the animals were traced using Ethovision XT 9 software (Noldus).

Decreased spontaneous alternation behavior in rodents is considered an animal model for perseverative symptoms in OCD patients^43^. Mice were placed in a Y-maze (Stoelting Co, Wood Dale, IL, USA) facing the wall of one of the arms (allocation to start arm was randomized), and allowed to explore for 5 min. Distal arm entries were identified using video tracing, and defined as the center point of the mouse being in the distal 1/3 of the arm. These distal arm entries were used to score spontaneous alternation behavior. An animal going back to the same distal arm after visiting the center triangle was scored as a repeated arm entry. Increased repeated arm entries are also indications of compulsive-like behavior.

The marble-burying test has been used as a model of compulsive-like and anxiety-like behaviors in mice, with an increased number of buried marbles reflecting increased compulsive-like behavior. Mice were placed individually in a clean home cage containing 18 glass marbles evenly spaced on 5 -cm deep sawdust, without access to food and water for 30 min. The number of marbles that were at least 2/3 buried reflects compulsive/anxiety-like digging behavior.

Upon entering an open field, mice are inclined to explore the peripheral border zone (thigmotaxis) and not the center zone of the maze. Mice were placed in the center of a home-built open field (55 × 55 cm) containing walls (40 cm height) and explored the maze freely for 30 min. A decrease in time spent in the center zone was used as an indication of anxiety-like behavior.

The elevated plus maze (EPM) is used to test anxiety in rodents^44^. Animals are placed at the junction of the four arms (two open and two closed arms) of the EPM (Stoelting Co), facing the open arm and allowed 5 min of free exploration. A decrease in time spent in the open arms and number of entries into the open arms reflects anxious behavior.

Following the behavioral studies, blood was collected via a tail vein puncture, and glucose levels were measured using an Accu-Check Aviva hand-held device (Roche Diabetes Care, Almere, The Netherlands).

### Magnetic resonance experiments

Magnetic resonance experiments were performed at an 11.7T BioSpec MR system (Bruker BioSpin, Ettlingen, Germany) equipped with an actively shielded gradient set of 600 mT/m. A circular polarized volume resonator and an actively decoupled mouse brain quadrature surface coil were used as transmit and receive coils, respectively, and data were acquired with Paravision 5.1 software. Mice were head-fixed in the magnet using a bite-bar and blunt earplugs and anesthetized by isoflurane. Anesthesia was induced with 3–4% isoflurane, after which it was maintained at ~2% (1:2 oxygen:air). The respiration rate of the mice was monitored throughout the experiment, and the animals’ temperature was maintained at 37.5 °C using a heated airflow device.

To visualize the brain anatomy, a T1-weighted gradient echo sequence in three orthogonal orientations was used. For diffusion tensor imaging (DTI) experiments, 20 axial slices covering the whole brain were acquired with a T2-weighted spin-echo echo-planar imaging (EPI) sequence^45^. Encoding b-factors of 0 (five b0 images) and 1000 s/mm^2^ were used, and diffusion sensitizing gradients were applied along 30 non-collinear directions with the following imaging parameters: TR = 7750 ms, TE = 21.4 ms, FOV = 20 × 20mm^2^, slice thickness = 0.5 mm, matrix = 128 × 128, Δ = 10 ms, δ = 4 ms, number of segments = 4, and acquisition time (TA) = 18 min.

Brain metabolite concentrations were quantified by single-voxel proton MRS using point resolved spectroscopy (PRESS) sequence (TR/TE = 2500/11.6 ms, 700 acquisitions and TA = 29 min) with image-guided positioning of voxels of 2.25 µL for the right dorsomedial striatum (DMS) and 1.47 µL for ACC in both hemispheres (Supplementary Fig. 1). Variable pulse power and optimized relaxation delays (VAPOR) were employed to suppress the water signal. For all spectra, a separate spectrum was acquired without suppressing the water signal, so that it could serve as a reference. We estimated the tissue levels of N-acetylaspartate (NAA), creatine (Cre), glutamate (Glu), glutamine (Gln), taurine (Tau), total choline (tCho), sum of myo-Inositol and glycine (mI + Gly), glucose (Glc), GABA, and glutathione (GSH).

### Euthanasia

Mice were euthanized by cervical dislocation, followed by collection of trunk blood and removal of the brain for proteomics. Blood was immediately centrifuged to obtain plasma. Brains and plasma were stored at −80 °C until further processing.

### Proteomics

The prefrontal cortex, the striatum, and the cerebellum were dissected from the frozen brains. Mouse brain tissues were weighed and, for each milligram of tissue, 10 μl of PBS (Sigma-Aldrich Company Ltd, Dorset, UK) with protease inhibitors (ThermoFisher, Waltham, MA, USA) was added. Tissues were lysed on ice by using a sonication probe and to each lysate, we added an equal volume of Tissue Extraction Reagent 1 (ThermoFisher) to that of PBS that was then vortexed. Lysates were centrifuged at 13,000 rpm for 3 min and then aliquoted and stored at −20 °C. Plasma samples were prepared as per kit protocols. Assays were performed based on the instructions provided in the manual of the analyte kits. Specifically, Kcnq1, Bdnf (ABIN2101758 and ABIN2115886, respectively, from antibodies-online GmbH, Aachen, Germany) and insulin (EMINS, ThermoFisher) were detected by means of ELISA, whereas Igf1 was assessed by the Luminex Multiplex kit (LXSAMSM-02, Bio-Techne Ltd, Abingdon, UK).

### Data analyses

Data from the behavior studies were analyzed using Ethovision XT 9 software (Noldus) for tracing. Unfortunately, several recordings from the open-field test were lost due to a technical failure, resulting in a lower power. In addition, two recordings from the EPM were excluded from further analyses, since the animals fell from the maze prior to the time limit. Tissue concentrations of MRS-detected metabolites were evaluated relative to the total creatine (tCr) signal, as it was found to be unchanged in our models when using water concentrations as reference and the metabolite’s signals were fitted by LCModel software^46^. Only signals with a Cramer-Rao lower bound (CRLB) ≤ 20% were included in the quantification. The DTI analysis was done using ExploreDTI^47^ through which fractional anisotropy (FA) and mean diffusivity (MD) maps were generated. Regions of interest (ROIs) were selected based on Allan mouse brain atlas^48^, and ImageJ software^49^ was used to draw ROIs on two subsequent slices (Supplementary Fig. 2) and extract their averaged FA and MD values.

Statistical analyses were performed using *IBM SPSS Statistics 22*. Due to large variation in the proteomics data, we applied Grubbs’ test^50^ for outlier correction to the data set. In addition, several analyses could not be executed due to either technical difficulties during the extraction of the tissue or due to limited tissue availability (see Supplementary Table 1 for details). Significance was tested via independent two-sided *t* tests (equal variances not assumed), and potential correlations were assessed by Pearson correlations. Both methods were then followed by correction for multiple testing using the false discovery rate (FDR) method, incorporating potential dependencies between *p*-values^51^. To calculate the FDR, we used the mafdr function in MATLAB (R2012a; The Mathworks) using the bootstrap selection method for the FDR parameter lambda. Whenever possible, the researchers were blinded with regard to the strain they were investigating. The data are displayed as mean ± SEM with *p* < 0.05 as significant.

## Results

### Phenotype of TALLYHO/JngJ mice

TH mice had increased blood glucose levels compared to SWR/J mice (332.1 ± 61.8 vs. 121.3 ± 5.7 mg/dl, *n* = 9 per strain, *p* = 0.009), confirming that they show glucose elevations consistent with DM2 (Supplementary Fig. 3A). TH mice demonstrated a significant reduction in locomotion, as quantified in the Y-maze (849.2 ± 113.2 vs. 1818.1 ± 36.6 cm traveled, *n* = 9 per strain, *p* = 0.000056), the open-field test (7698.7 ± 767.0 and 12952.5 ± 903.4 cm traveled, *p* = 4 per strain, *p* = 0.005) and the EPM (512.0 ± 95.5 vs. 1388.5 ± 33.8 cm traveled, *n* = 7 [TH] or *n* = 9 [SWR/J], *p* = 0.000036) (Supplementary Fig. 3B). These findings are in keeping with known literature^40,52^.

### TALLYHO/JngJ mice are more perseverative and anxious

**Fig. 1:**
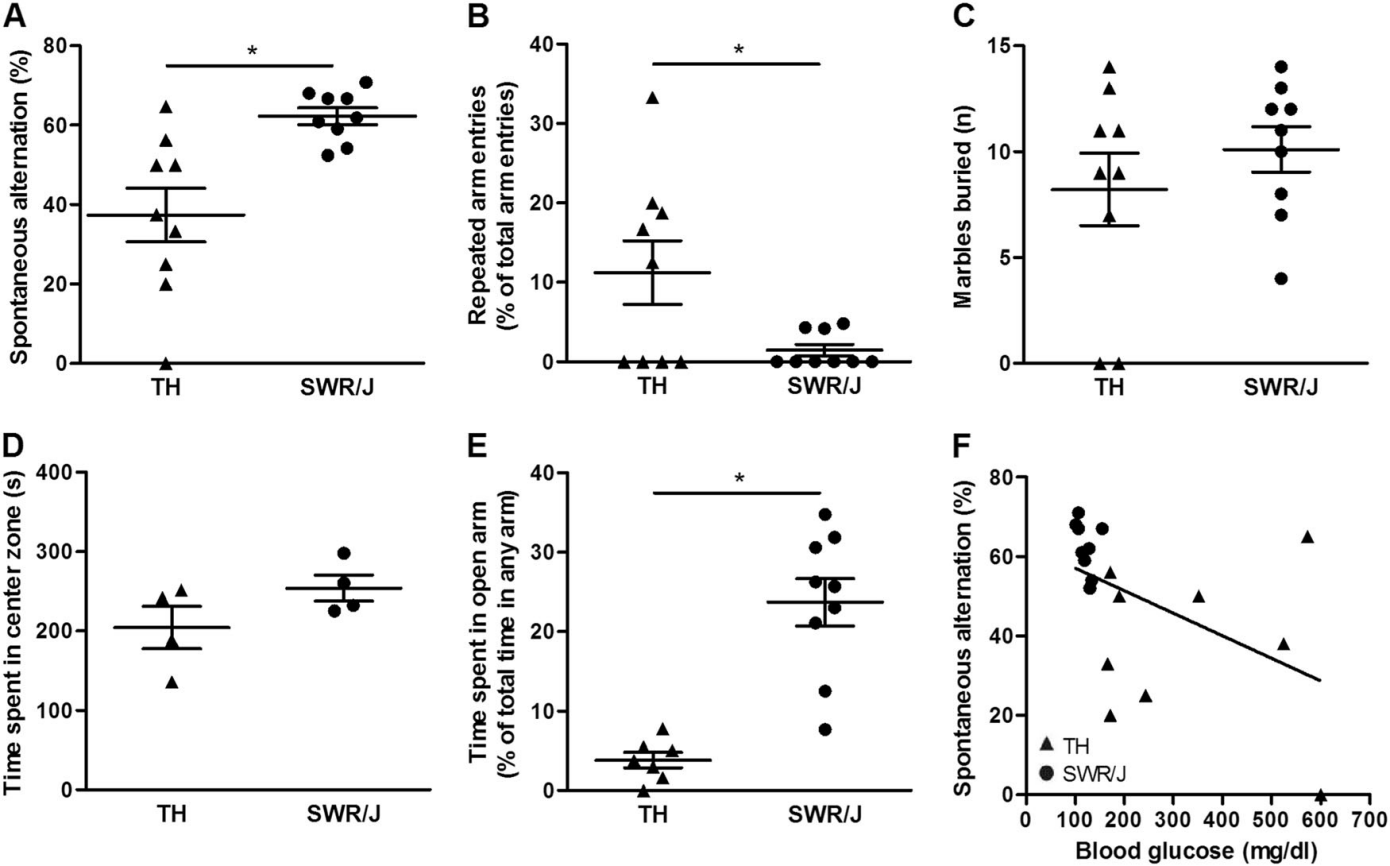
Increased compulsive- and anxiety-like behaviours are observed in TALLYHO/JngJ mice while compulsive behaviour correlates with blood glucose levels. **a** TALLYHO/JngJ (TH) mice show increased compulsive behavior compared to SWR/J mice (the control strain), as seen in a decrease in the spontaneous alternation behavior (37.4 ± 6.8 vs. 62.3 ± 2.1% spontaneous alternation, *n* = 9 per strain, *p* = 0.006). **b** In addition, an increase in the number of repeated arm entries was observed in the TH mice (11.3 ± 4.0 vs. 1.48 ± 0.74% of the total arm entries being repeated arm entries, *n* = 9 per strain, *p* = 0.041). **c** No difference was observed between the two strains in compulsive marble burying (8.2 ± 1.7 vs. 10.1 ± 1.1 marbles buried, *n* = 9 per strain, *p* = 0.365). **d** In addition, TH mice display increased anxiety behavior: a trend was observed toward a TH mice spending less time in the center zone of the open field compared with SWR/J mice (204.6 ± 26.6 vs. 254.2 ± 16.5 s, *n* = 4 per strain, *p* = 0.174). **e** More importantly, TH mice spent less time in the open arms of the elevated plus maze (EPM), suggesting increased anxiety (3.8 ± 0.98 versus 23.7 ± 2.97 s, *n* = 7 [TH] and *n* = 9 [SWR/J], *p* = 0.000096). **f** Blood glucose levels were negatively correlated with spontaneous alternation behavior (*r* = −0.495, *n* = 18, *p* = 0.037)

Decreased spontaneous alternation behavior reflects compulsivity-like behavior^43^. When allowed to explore freely, mice alternate entering the three arms of the Y-maze. TH mice showed a considerable reduction in spontaneous alternation behavior (37.4 ± 6.8 vs. 62.3 ± 2.1% spontaneous alternation, *n* = 9 per strain, *p* = 0.006; Fig. 1a). In addition, TH mice frequently entered the same arm twice successively (11.3 ± 4.0 vs. 1.48 ± 0.74% of the total arm entries being repeated arm entries, *n* = 9 per strain, *p* = 0.041; Fig. 1b). No compulsive digging was observed in the marble-burying test (8.2 ± 1.7 vs. 10.1 ± 1.1 marbles buried, *n* = 9 per strain, *p* = 0.365; Fig. 1c). We also tested whether TH mice exhibited altered anxiety levels. In the open field, there was a trend toward TH mice spending less time in the center (204.6 ± 26.6 vs. 254.2 ± 16.5 s, *n* = 4 per strain, *p* = 0.174; Fig. 1d). More importantly, TH mice spent less time in the open arms of the EPM, suggesting increased anxiety (3.8 ± 0.98 vs. 23.7 ± 2.97 s, *n* = 7 [TH] and *n* = 9 [SWR/J], *p* = 0.000096; Fig. 1e). Interestingly, blood glucose levels were negatively correlated with spontaneous alternation behavior (*r* = −0.495, *n* = 18, *p* = 0.037; Fig. 1f), suggesting that higher blood glucose levels (i.e. hyperglycemia) was associated with an increase in compulsive-like behavior. Taken together, TH mice present both compulsivity-like and anxious behaviors.

### MRS reveals changes in the ACC and DMS in TALLYHO/JngJ mice

**Fig. 2:**
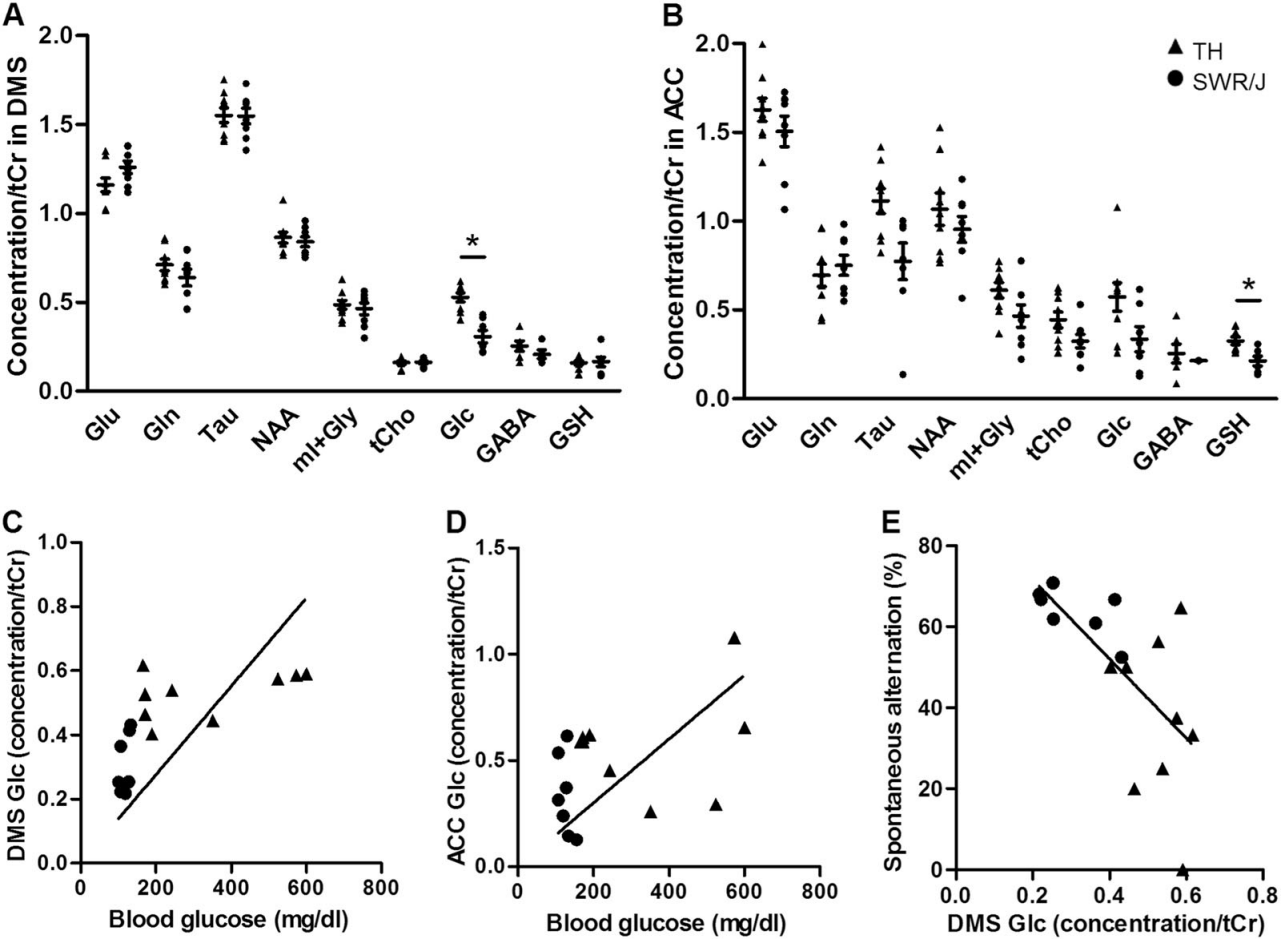
MRS revealed differences in the metabolite content of the dorsomedial striatum (DMS) and anterior cingulate cortex (ACC) in TALLYHO/JngJ mice. The metabolites of interest are glutamate (Glu), glutamine (Gln), taurine (Tau), N-acetylaspartate (NAA), sum of myo-Inositol and glycine (mI + Gly), total choline (tCho), glucose (Glc), GABA, and glutathione (GSH). MRS data are expressed as ratios of the metabolites relative to the total creatine (tCr) levels. **a** Glucose (Glc) ratios (relative to tCr) were increased in brains of TALLYHO/JngJ (TH) mice in the DMS compared with the control strain (SWR/J mice) (0.527 ± 0.025 vs. 0.308 ± 0.035, *n* = 9 [TH] and *n* = 7 [SWR/J], *p* = 0.0031). **b** An increase in glutathione (GSH) ratios (relative to tCr) was observed in the ACC of TH mice (0.325 ± 0.021 and 0.212 ± 0.028, *n* = 9 [TH] and *n* = 6 [SWR/J], *p* = 0.047). Differences in the other metabolites (glutamate [Glu] glutamine [Gln], taurine [Tau], N-acetylaspartate [NAA], the sum of myo-Inositol and glycine [mI + Gly], total choline [tCho], and GABA) failed to reach significance. **c** Blood glucose concentrations correlated with DMS Glc concentration (*r* = 0.664, *n* = 16, *p* = 0.005). **d** No significant correlation was observed between the blood glucose concentrations and ACC Glc concentration (*r* = 0.415, *n* = 16, *p* = 0.11). **e** Interestingly, we observed a significant negative correlation between glucose levels in the DMS specifically, and spontaneous alternation (*r* = −0.658, *n* = 16, *p* = 0.006)

Single-voxel proton MRS was performed to assess metabolite levels in ACC and DMS of the TH and SWR/J mice. The metabolites are reported relative to tCr as it was found to be unchanged in our models when using water concentrations as reference (DMS: 19.7 ± 1.76, *n* = 9 [TH] and 19.6 ± 0.61, *n* = 8 [SWR/J], *p* = 0.95; ACC: 12.8 ± 1.70, *n* = 9 [TH], 14.0 ± 2.74, *n* = 6 [TH], *p* = 0.70, Supplementary Fig. 4). The TH mice had elevated Glc levels in the DMS (0.527 ± 0.025 vs. 0.308 ± 0.035, *n* = 9 [TH] and *n* = 7 [SWR/J], *p* = 0.0031; Fig. 2a). In addition, GSH levels were specifically increased in the ACC of TH mice (0.325 ± 0.021 and 0.212 ± 0.028, *n* = 9 [TH] and *n* = 6 [SWR/J], *p* = 0.047; Fig. 2b). Glc levels in the DMS (*r* = 0.664, *n* = 16, *p* = 0.005) but not in the ACC (*r* = 0.415, *n* = 16, *p* = 0.11) correlated with their concentration in blood (Fig. 2c, d). In addition, we observed a significant negative correlation between glucose levels in the DMS specifically, and spontaneous alternation (*r* = −0.658, *n* = 16, *p* = 0.006; Fig. 2e and Supplementary Table 2), suggesting that an increase in the glucose levels in the DMS resulted in increased compulsivity-like behavior.

### White matter microstructure changes may underlie compulsivity-like behavior

**Fig. 3:**
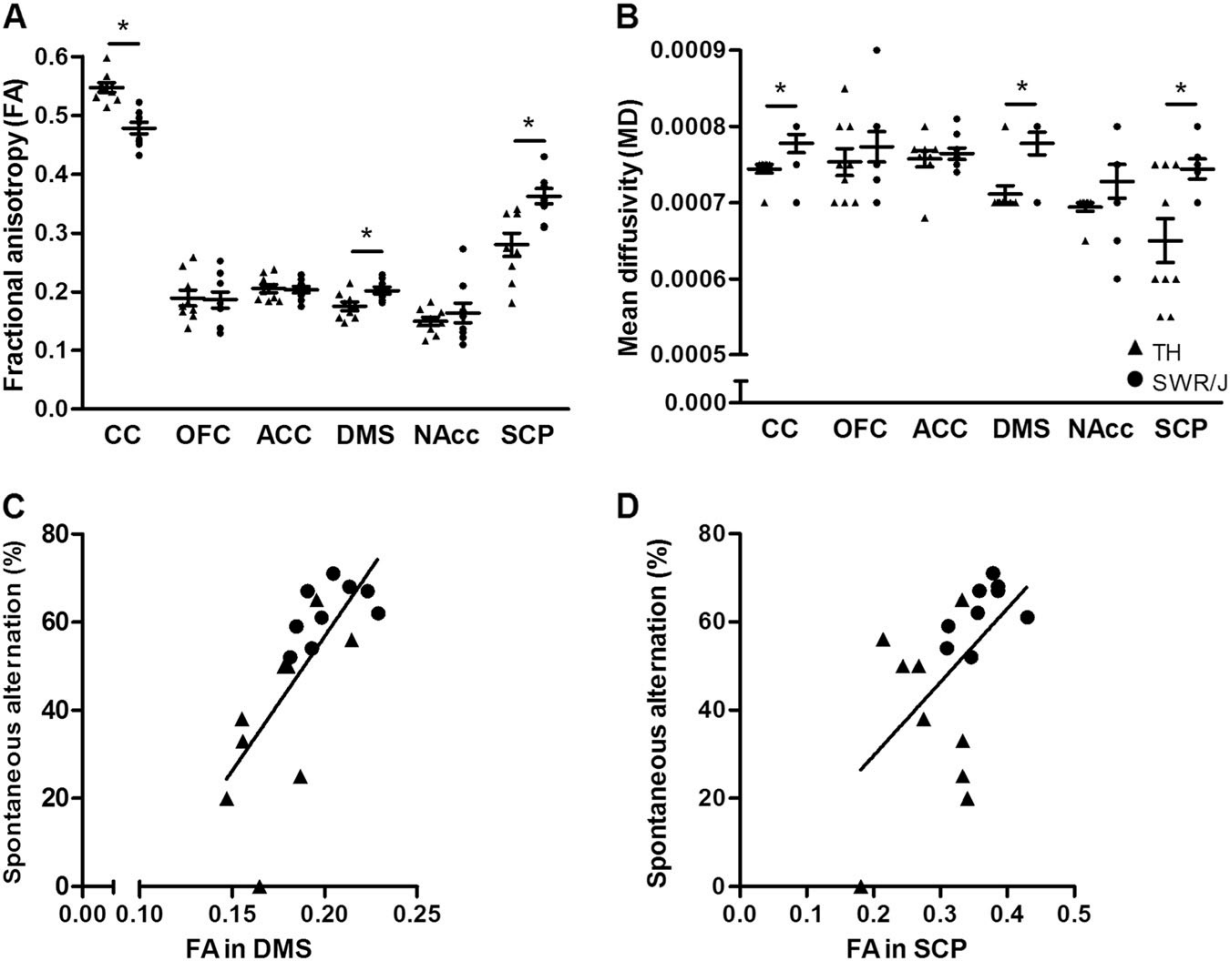
DTI showed differences in white matter microstructure in brains of TALLYHO/JngJ (TH) mice compared to the control strain (SWR/J). **a** The fractional anisotropy (FA) was increased in the corpus callosum (CC) (0.547 ± 0.008 vs. 0.478 ± 0.01, *n* = 9 per strain, *p* = 3.7E-05) and decreased in the dorsomedial striatum (DMS) (0.18 ± 0.01 vs. 0.20 ± 0.01, *n* = 9 per strain, *p* = 0.01) and superior cerebellar peduncle (SCP) (0.28 ± 0.02 vs. 0.36 ± 0.01, *n* = 9 per strain, *p* = 0.003) of TH mice. No differences were observed in the orbitofrontal cortex (OFC), anterior cingulate cortex (ACC), and nucleus accumbens (NAcc). **b** The same three brain regions showed a decrease in the mean diffusivity (MD) in TH mice: CC (7.44E-04 ± 5.56E-06 vs. 7.78E-04 ± 1.21E-05, *n* = 9 per strain, *p* = 0.03), DMS (7.17E-04 ± 1.10E-05 vs. 7.58E-04 ± 1.18E-05, *n* = 9 per strain, *p* = 0.02) and SCP (6.50E-04 ± 2.89E-05 vs. 7.44E-04 ± 1.30E-05, *n* = 9 per strain, *p* = 0.01). **c**, **d** Compulsivity-like behavior as shown by the spontaneous alternation is positively correlated to the FA in DMS and SCP (*r* = 0.734, *n* = 18, *p* = 0.002 and *r* = 0.548, *n* = 18, *p* = 0.02, respectively)

The FA and MD of the corpus callosum (CC), OFC, ACC, DMS, nucleus accumbens (NAcc), and the superior cerebellar peduncles (SCP) were assessed (Supplementary Fig. 5). TH mice had increased FA (0.547 ± 0.008 vs. 0.478 ± 0.01, *n* = 9 per strain, *p* = 3.7E-05) and decreased MD (7.44E-04 ± 5.56E-06 vs. 7.78E-04 ± 1.21E-05, *n* = 9 per strain, *p* = 0.03) in the CC, decreased FA (0.18 ± 0.01 vs. 0.20 ± 0.01, *n* = 9 per strain, *p* = 0.01) and MD (7.17E-04 ± 1.10E-05 vs. 7.58E-04 ± 1.18E-05, *n* = 9 per strain, *p* = 0.02) in the DMS, and decreased FA (0.28 ± 0.02 vs. 0.36 ± 0.01, *n* = 9 per strain, *p* = 0.003) and MD (6.50E-04 ± 2.89E-05 vs. 7.44E-04 ± 1.30E-05, *n* = 9 per strain, *p* = 0.01) values in the SCP (Fig. 3a, b). Of note, FA in the DMS and the SCP of TH and SWR/J animals positively correlated with spontaneous alternation behavior (*r* = 0.734, *n* = 18, *p* = 0.002 and *r* = 0.548, *n* = 18, *p* = 0.02, respectively), suggesting that the increased FA in these brain regions is associated with increased spontaneous alternation, irrespective of the mouse strain (Fig. 3c, d). Correlations between other DTI findings and spontaneous alternation behavior failed to reach significance (Supplementary Table 3). In addition, we performed correlation analyses between the DTI and MRS results in the corresponding brain regions, but all correlations failed to reach significance (Supplementary Table 4).

### Levels of Insulin-like growth factor 1 proteins in TALLYHO/JngJ mice are altered in both plasma and brain

**Fig. 4:**
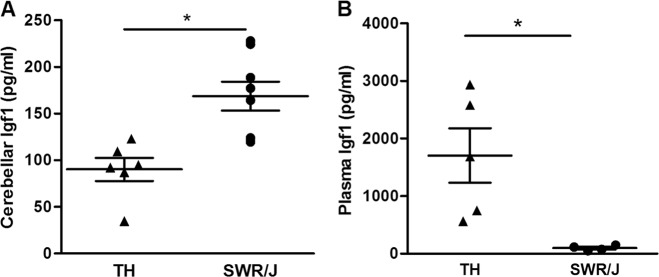
TALLYHO/JngJ mice show changes in cerebellar and plasma Insulin-like growth factor 1 protein levels. **a** The concentration of Insulin-like growth factor 1 (Igf1) is reduced in the cerebellum of TALLYHO/JngJ (TH) mice compared with their controls, SWR/J mice (90.2 ± 12.3 vs. 168.7 ± 15.5 pg/ml, *n* = 6 [TH] and *n* = 8 [SWR/J], *p* = 0.0002). **b** Interestingly, the concentration of Igf1 is increased in the blood plasma of TH mice (1217.4 ± 453.6 vs. 97.6 ± 22.2 pg/ml, *n* = 7 [TH] and *n* = 4 [SWR/J], *p* = 0.049)

Protein expression was examined for four molecules that have been associated with OCD before: insulin^18^, insulin-like growth factor 1 (Igf1)^18,53^, potassium voltage-gated channel subfamily KQT member 1 (Kcnq1)^18^—that inhibits insulin secretion^54^—and brain-derived neurotrophic factor (Bdnf)—a positive regulator of insulin secretion and known to be involved in OCD^18,55–58^. Protein expression levels were determined in the plasma, prefrontal cortex, striatum, and cerebellum of TH and SWR/J mice (Supplementary Table 1). The protein selection was based on the molecular landscape of OCD that we built (see above)^18^. Igf1 concentrations were decreased in the cerebellum of TH mice (90.2 ± 12.3 vs. 168.7 ± 15.5 pg/ml, *n* = 6 [TH] and *n* = 8 [SWR/J], *p* = 0.0002; Fig. 4a), while they were increased in the plasma of these mice (1217.4 ± 453.6 vs. 97.6 ± 22.2 pg/ml, *n* = 7 [TH] and *n* = 4 [SWR/J], *p* = 0.049, Fig. 4b). In all tested samples, insulin and Bdnf expression in plasma fell below the detection limit, which was also the case for Igf1 levels in the striatum. No significant correlations between the proteomic data set and OCD-like behavior were observed (Supplementary Table 5).

## Discussion

Previously, we proposed a role for (CNS) insulin-regulated dendritic spine formation and synaptic plasticity in OCD in a molecular landscape based on data from genome-wide association studies^18^. The aim of this study was to find additional evidence for the involvement of insulin-related signaling in OCD, which was carried out at three levels: behavioral assessment of TH mice (a model for DM2), characterization of their brains via MR imaging and spectroscopy and investigation of the prefrontal cortex, striatum, cerebellum, and plasma of these mice at the molecular level.

At the behavioral level, we showed that TH mice display a compulsivity-like phenotype, and that these mice were more anxious than their control counterparts. These results are in line with previously published findings that anxiety is a well-known symptom of OCD^1^. In addition, patients with DM1 and DM2 display more symptoms of OCD than control subjects^22,23^, and the prevalence of anxiety symptoms in patients with diabetes is higher than in the general population^59^.

Regarding the characterization of the brain, MRS revealed an increase in glucose levels in the DMS. In this respect, insulin resistance inherent to DM2 may have caused increased glucose levels in the DMS, which suggests that DM2 in TH animals has a similar effect on the glucose metabolism of the DMS than on the peripheral circulation. In turn, this is in keeping with our finding that both blood and DMS glucose levels are negatively correlated with spontaneous alternation behavior. This also provides a more direct link between CNS glucose levels and OCD-like behavior, i.e., increased CNS glucose is associated with increased OCD-like behavior. That being said, it is still unclear what is the relative contribution of this change in glucose metabolism in the periphery and the CNS at the behavioral level.

The second finding of our MRS analyses is an increase in GSH in the ACC of TH mice. GSH has previously been studied in the context of OCD, although the number of studies is limited. Although no studies show changes in the anterior cingulate cortex, GSH levels in the posterior cingulate cortex were found to be significantly lower in OCD patients vs. control subjects^60^. In addition, a reduction in GSH levels in the serum of OCD patients was reported^61^, suggesting that GSH levels are affected in both the CNS and peripheral tiisues of OCD patients. This is corroborated by findings of altered GSH levels in animal models of OCD: *Sapap3* knockout mice show a reduction in GSH levels in the striatum^62^, and deer mice that show high levels of stereotypical behavior have reduced GSH levels in the frontal cortex^63^.

Interestingly, in keeping with the main aim of this study (see above) and although more research is warranted, our finding about GSH also adds to the evidence about insulin signaling being implicated in OCD-like behavior. Insulin itself stimulates the synthesis of GSH^64^, while GSH is also involved in the same PI3K/AKT/RAC1 signaling cascades that are regulated by insulin^18^. For instance, GSH inhibits the activation of RAC1^65^ whereas activation of PI3K and AKT regulates GSH synthesis^64,66^. In summary, our MRS results provide further insights and clues for further research into how insulin regulates OCD-linked behavior by affecting specific brain regions. However, the relative contribution of the direct metabolic effects and indirect effects of insulin on synaptic plasticity needs to be elucidated.

On the level of the white matter microstructure, DTI revealed TH mice displaying differences in the CC, DMS, and SCP. Our finding of changes in the CC is in line with previous studies, since multiple studies report increased FA in the CC in OCD patients, but other studies found a decreased FA in this brain region, both in adult and pediatric populations (reviewed in ref. ^67^), suggesting that although no consensus has been reached about the directionality of the effect, it is clear that the white matter microstructure of the CC is affected in OCD patients. Of particular notice is one study, in which drug-naive OCD patients were shown to have an increase in FA in the CC, the internal capsule and white matter in the area superolateral to the right caudate^68^. This increase in FA was no longer observed after 12 weeks of citalopram treatment^68^. Lastly, although we found no significant correlation between FA in the CC and spontaneous alternation behavior, it is interesting to note that FA reduction in the CC was found to be associated with greater insulin resistance in generally healthy adults^36^, providing a clue as to how FA changes may be related to disturbed insulin signaling. Few studies have investigated the white matter microstructure in the DMS and/or the SCP of OCD patients, and no consensus has been reached regarding these differences^69–71^. Of note, one recent study found an increase in FA in the cerebellum of OCD patients^72^, which is in line with our finding of increased FA in the SCP. In addition, although white matter microstructure is known to be altered in DM1 and DM2^38,39^, no studies have shown differences in specifically the CC, DMS, or SCP in patients with DM2.

In addition, DTI analyses revealed that the FA of the DMS and SCP positively correlated with spontaneous alternation behavior in the Y-maze. This correlation may indicate that differences in white matter microstructure at least partially underlie the compulsivity-like behavior we observed. Most interestingly, our finding of a positive correlation between glucose levels in the blood—which are regulated by peripheral insulin—and in the DMS (see above), suggests an “overspill” of blood glucose into the DMS. Combined with the observed positive correlation between FA in the DMS and spontaneous alternation behavior, this suggests that increased DMS glucose increases compulsivity-like behavior through decreasing FA. Taken together, all these findings indicate that at least to some extent, compulsive behavior is indirectly regulated by peripheral insulin signaling.

At the molecular level, we found no significant changes in the concentrations of insulin, Kcnq1, and Bdnf in any of the investigated tissues. However, it is important to note that insulin-related signaling involves a wide variety of molecules, some of which we did find to be significantly changed in the TH animals. In this respect, we observed that TH mice have a decreased expression of Igf1 in the cerebellum, but increased Igf1 plasma levels. Our findings of different Igf1 levels in the cerebellum and plasma of compulsive TH mice vs. controls support our hypothesis that insulin-related signaling pathways are involved in compulsive behavior, possibly affecting SCP microstructure. Specifically, the observed imbalance between peripheral and central Igf1 expression is noteworthy. First, IGF1 levels were found to be increased in the serum of OCD patients compared with controls^53^, which is in line with the increased plasma Igf1 levels we observed in the TH mice. Disturbances in IGF1 levels such as that we observed may be an indication of reduced sensitivity of tissues to IGF1, so called IGF1 resistance, which often accompanies insulin resistance in DM2^73^. Insulin/IGF1 resistance blunts the activation of the insulin receptor and IGF1 receptor (IGF1R) signaling cascades, which could negatively impact on dendritic spine and synapse formation^21^.

Interestingly, IGF1 is also produced locally in the cerebellum, and can act there in a paracrine or autocrine fashion^74^. IGF1 and IGF1R are known to be abundant in the cerebellum both in rodents and humans^75,76^. More specifically, IGF1R is present both presynaptically (i.e. in axonal terminals making contact with the soma of cerebellar Purkinje cells) and postsynaptically, in the dendrites and soma of Purkinje cells of the cerebellar cortex^76^. IGF1 is extremely important during the (pre- and postnatal) development of the cerebellum, as it is essential for normal dendritic growth and Purkinje cell survival^74,77^ and for regulating synaptic plasticity of the cerebellum in general^78^. Given the above, we would like to speculate that the observed imbalance in Igf1 expression levels in TH animals (i.e., more Igf1 in plasma and less Igf1 in cerebellum) leads to compulsive behavior because the decrease of available Igf1 to bind Igf1r has a negative impact on synaptic plasticity in the cerebellum and subsequently on spontaneous alternation behavior.

Although no studies have been undertaken to elucidate the effects of dendritic spine and synapse formation in human subjects, this is well studied in animal models of OCD. *Hoxb8* mutant mice—that exhibit compulsive grooming similar to humans with OCD-like traits—display profound differences in synaptic plasticity^79^. In addition, mice that lack SAP90/PSD95-associated protein 3 (*Sapap*3; also known as *Dlgap*3), a postsynaptic scaffolding protein at excitatory synapses that is highly expressed in the striatum, exhibit increased anxiety and compulsive grooming behavior combined with defects in corticostriatal synapses^80^. Specifically, these effects are caused by a reduction in the AMPA-type glutamate receptor (AMPAR)-mediated synaptic transmission in corticostriatal synapses^81,82^.

Taken together, our experimental findings support our hypothesis that insulin-related pathways are involved in OCD etiology while also supporting an association between white matter integrity and compulsive behavior. Although it is still not clear what the relative contribution is of (1) the effect of ‘spill over’ peripheral insulin and (2) locally produced CNS insulin, our data suggest that at least some of the observed behavioral changes are due to the effects of CNS insulin. That being said, further research is required in order to dissociate the peripheral and central insulin effects. For example, the effect on OCD(-like symptoms) could be tested by using pharmacological interventions such as an IGF1 agonist or metformin, a first line treatment of DM2. In addition, mouse models with a conditional/brain region-specific knockdown of *Igf1* and/or *Igf1r* could be behaviorally assessed.

## Supplementary information

Supplementary Figure 1

Supplementary Figure 2

Supplementary Figure 3

Supplementary Figure 4

Supplementary Figure 5

Supplementary Table 1

Supplementary Table 2

Supplementary Table 3

Supplementary Table 4

Supplementary Table 5
