# Supplementary material for: Converging evidence points towards a role of insulin signaling in regulating compulsive behavior
Source: Transl Psychiatry. 2019 Sep 12;9:225. doi: 10.1038/s41398-019-0559-6 (PMC6742634; doi:10.1038/s41398-019-0559-6)
Supplement: Supplementary file 1 — Supplementary Figure 1 [file 41398_2019_559_MOESM1_ESM.docx]

**Supplementary Figure 1: Representative spectrum and voxel localization**
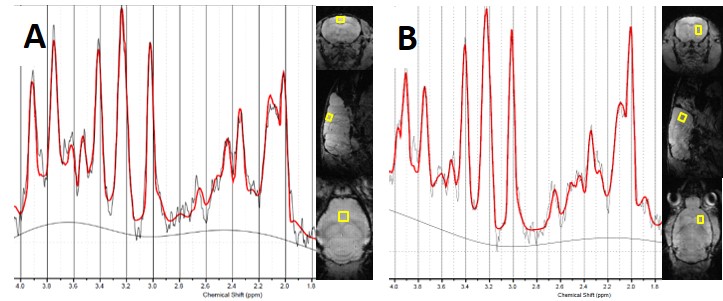


**Supplementary Figure 1.** Representative spectrum and voxel localization in the (A) anterior cingulate cortex (ACC) and (B) dorsomedial striatum (DMS).
