# Supplementary material for: Converging evidence points towards a role of insulin signaling in regulating compulsive behavior
Source: Transl Psychiatry. 2019 Sep 12;9:225. doi: 10.1038/s41398-019-0559-6 (PMC6742634; doi:10.1038/s41398-019-0559-6)
Supplement: Supplementary file 2 — Supplementary Figure 2 [file 41398_2019_559_MOESM2_ESM.docx]

**Supplementary Figure 2: Definition of regions of interest in DTI analysis**

**Supplementary Figure 2.** Regions of interest were drawn using the following landmarks: (A) corpus callosum defined as below the restrosplenial and secondary motor area and the above the stratum radiatum; (B) anterior cingulate cortex defined as above the corpus callosum and cingulum bundle, and enclosed by the secondary motor area; (C) the orbitofrontal cortex defined as below the primary and secondary motor area and anterior cingulate cortex, enclosed by the infralimbic agranular insular area and above the olfactory areas; (D) the dorsal medial striatum defined as below the corpus callosum and lateral ventricle, enclosed by the external capsule and endopiriform nucleus and above the nucleus accumbens and medial to the anterior commissure; (E) the nucleus accumbens defined as below the dorsomedial striatum, anterior commissure and nuclei of the stria terminalis, enclosed by the piriform and preoptic area, and above the substantia innominata; and (F) the superior cerebellar peduncles defined as below lobules II and III, and enclosed by the parabrachial nucleus and arbor vitae.
