# Supplementary material for: Converging evidence points towards a role of insulin signaling in regulating compulsive behavior
Source: Transl Psychiatry. 2019 Sep 12;9:225. doi: 10.1038/s41398-019-0559-6 (PMC6742634; doi:10.1038/s41398-019-0559-6)
Supplement: Supplementary file 3 — Supplementary Figure 3 [file 41398_2019_559_MOESM3_ESM.docx]

**Supplementary Figure 3: Phenotype of TH mice**


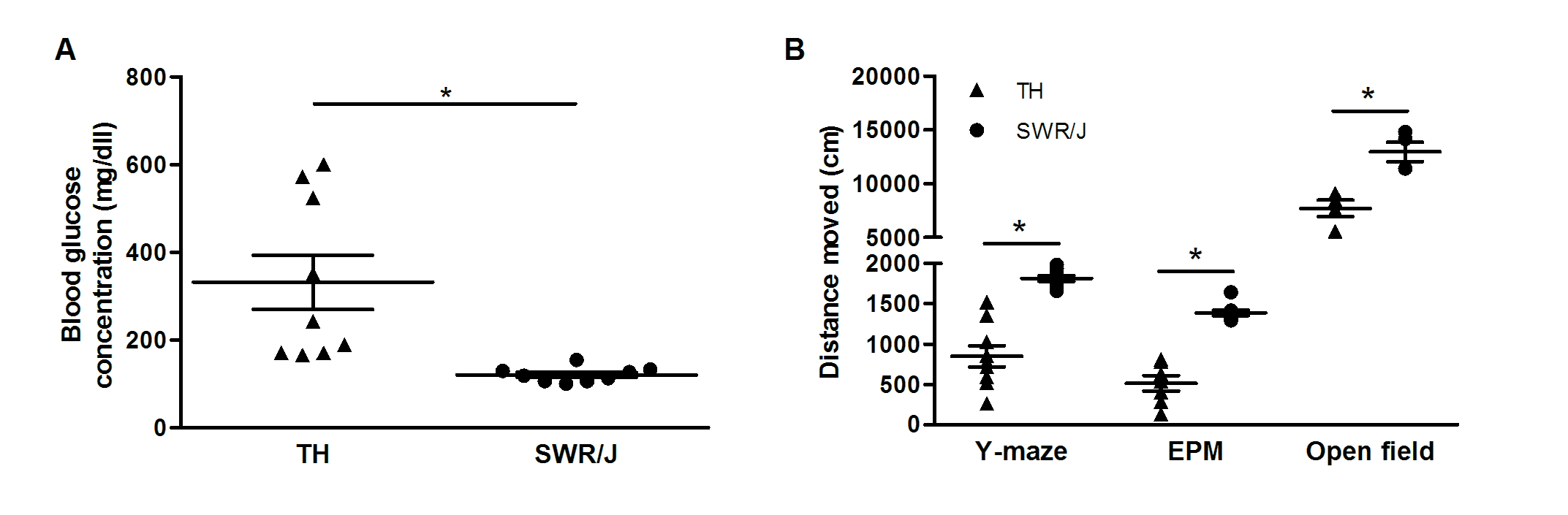


**Supplementary Figure 3.** TALLYHO/JgnJ (TH) mice (n=9) had increased blood glucose levels (p=0.009) compared to their control strain, the SWR/J mice (n=9; 332.1±61.8 and 121.3±5.7 mg/dl, respectively), indicating that they developed DM2 (**A**). Additionally, TH mice showed a significant reduction in locomotion observed in three behavioral assays. In the Y-maze, TH mice (n=9) travelled 849.2±113.2 cm, whereas SWR/J mice (n=9) moved over a distance of 1818.1±36.6 cm (p=0.000056). In the evelated plus maze (EPM), TH mice (n=7) walked 512.0±95.5 cm and SWR/J mice (n=9) walked for 1388.5±33.8 cm (p=0.000036). Lastly, in the open field the TH mice (n=4) and SWR/J mice (n=4) travelled 7698.7±767.0 and 12952.5±903.4 cm, respectively (p=0.005) (**B**).
