# Supplementary material for: Converging evidence points towards a role of insulin signaling in regulating compulsive behavior
Source: Transl Psychiatry. 2019 Sep 12;9:225. doi: 10.1038/s41398-019-0559-6 (PMC6742634; doi:10.1038/s41398-019-0559-6)
Supplement: Supplementary file 4 — Supplementary Figure 4 [file 41398_2019_559_MOESM4_ESM.docx]

**Supplementary Figure 4: Total creatine levels were unchanged in both strains**


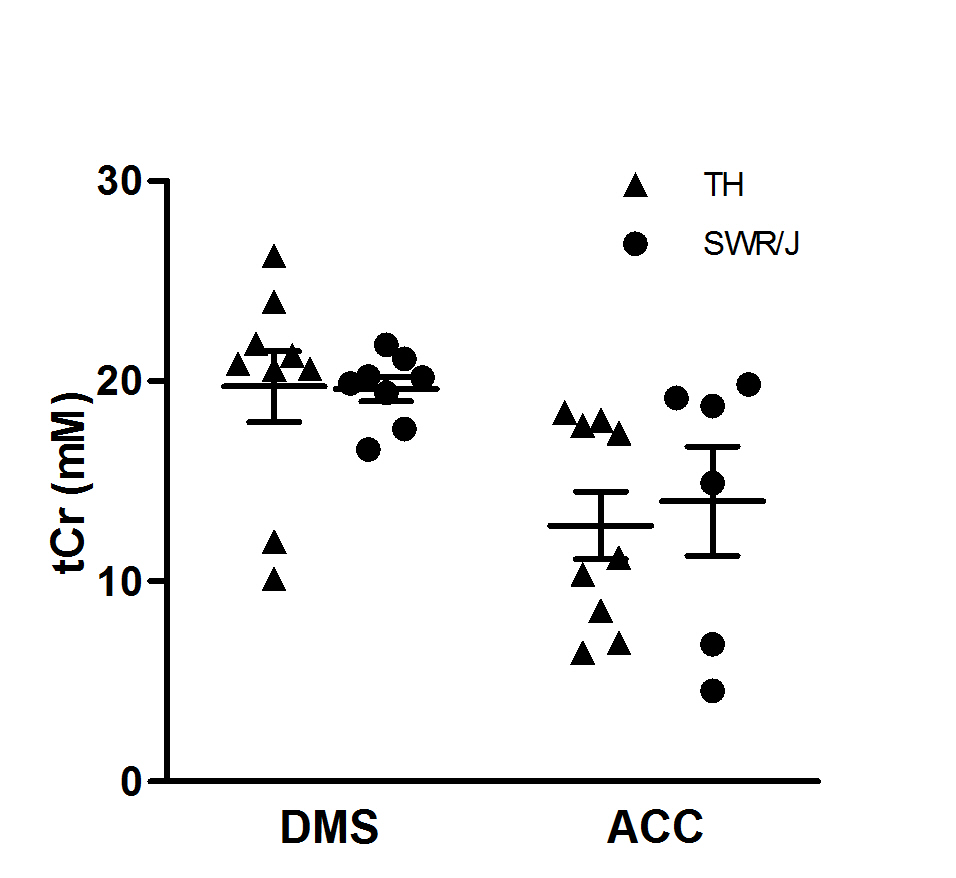


**Supplementary Figure 4.** Single-voxel MRS was performed to assess metabolite levels in dorsomedial striatum (DMS) and anterior cingulate cortex (ACC) of the TALLYHO/JngJ (TH) mice and the control strain, SWR/J mice. The metabolites are reported relative to total creatine (tCr) as it was found to be unchanged in our models when using water concentrations as reference (DMS: 19.7±1.76, n=9 [TH] and 19.6±0.61, n=8 [SWR/J], p=0.95; ACC: 12.8±1.70, n=9 [TH], 14.0±2.74, n=6 [TH], p=0.70.
