# Supplementary material for: Converging evidence points towards a role of insulin signaling in regulating compulsive behavior
Source: Transl Psychiatry. 2019 Sep 12;9:225. doi: 10.1038/s41398-019-0559-6 (PMC6742634; doi:10.1038/s41398-019-0559-6)
Supplement: Supplementary file 5 — Supplementary Figure 5 [file 41398_2019_559_MOESM5_ESM.docx]

**Supplementary Figure 5: FA, MD and FEFA maps of TH and SWR/J brains**

**Supplementary Figure 5.** Fractional Anisotropy (FA), Mean Diffusivity (MD) and First Eigenvector Fractional Anisotropy (FEFA) maps for the SWR/J controls and TALLYHO/JngJ (TH) mice. In the FEFA maps, each voxel is given a color representing the direction of its first eigenvector to display the anisotropy direction/orientation of the brain.
