# Supplementary material for: Converging evidence points towards a role of insulin signaling in regulating compulsive behavior
Source: Transl Psychiatry. 2019 Sep 12;9:225. doi: 10.1038/s41398-019-0559-6 (PMC6742634; doi:10.1038/s41398-019-0559-6)
Supplement: Supplementary file 6 — Supplementary Table 1 [file 41398_2019_559_MOESM6_ESM.docx]

**Supplementary Table 1: Outlier analysis proteomic dataset**

| **Supplementary Table 1:** Protein expression levels (pg/ml) of insulin, insulin-like growth factor 1 (Igf1), potassium voltage-gated channel subfamily KQT member 1 (Kcnq1) and brain-derived neurotrophic factor (Bdnf) were assessed in the prefrontal cortex, striatum, cerebellum and blood plasma of TALLYHO/JngJ (TH) (n=7) and control SWR/J (n=9) mice. Data are displayed as mean (SEM). N.D. indicates that the analysis could not be performed for this type of tissue or protein due to limitations in the availability of the tissue. N.A. is an indicator that the tissue was not available for analysis due to technical problems while obtaining the tissue. Outliers assessed via Grubbs' test are indicated with an asterisk (*) and not taken into account for subsequent analyses. The expression data was analyzed using individual T-tests, and corrected for multiple testing using the False Discovery Rate (FDR) method. The expression data is displayed as mean (SEM). | | | | | | | | | | |
| --- | --- | --- | --- | --- | --- | --- | --- | --- | --- | --- |
| **Tissue of interest** | | **Insulin (pg/ml)** | | | **Igf1 (pg/ml)** | | **Kcnq1 (pg/ml)** | | **Bdnf (pg/ml)** | |
|  |  | **TH** | **SWR/J** | | **TH** | **SWR/J** | **TH** | **SWR/J** | **TH** | **SWR/J** |
| **Prefrontal cortex** | **Sample 1** | 28.1 | 17.6 | | 42.8 | 46.1 | 10950.0 | 11602.5 | 1897.5 | 1517.8 |
|  | **Sample 2** | 15.6 | 13.3 | | 44.4 | 47.7 | 13485.0 | 15015.0 | 1717.5 | 1593.8 |
|  | **Sample 3** | 13.7 | 35.8 | | N.D. | 55.3 | 11370.0 | 11055.0 | 1680.9 | 1686.6 |
|  | **Sample 4** | 21.1 | 24.5 | | N.D. | 56.9 | 12697.5 | 12922.5 | 1600.3 | 1954.7 |
|  | **Sample 5** | 39.7 | 28.8 | | 67.7 | 41.7 | 14430.0 | 11137.5 | 1556.3 | 1707.2 |
|  | **Sample 6** | 12.2 | 15.8 | | 44.4 | 98.6 | 11505.0 | 11017.5 | 1456.9 | 2063.4 |
|  | **Sample 7** | 32.0 | 13.1 | | 54.5 | N.D. | 10005.0 | 13050.0 | 1673.4 | 1939.7 |
|  | **Sample 8** | N.A. | 16.9 | | N.A. | 65.0 | N.A. | 13950.0 | N.A. | 1480.3 |
|  | **Sample 9** | N.A. | 24.1 | | N.A. | 50.4 | N.A. | 10357.5 | N.A. | 1481.3 |
|  | **Mean (SEM)** | **23.2 (3.9)** | **21.1 (2.6)** | | **50.7 (4.7)** | **57.7 (6.4)** | **12063.2 (583.4)** | **12234.2 (524.5)** | **1654.7 (52.5)** | **1713.9 (73.9)** |
|  | **N** | **7** | **9** | | **5** | **8** | **7** | **9** | **7** | **9** |
|  | **P-value** | **0.66** | | | **0.40** | | **0.83** | | **0.53** | |
| **Striatum** | **Sample 1** | **35.7** | | **29.7** | **N.D.** | **N.D.** | **13027.5** | **12030.0** | **2595.9** | **2124.4** |
|  | **Sample 2** | **11.9** | | **21.3** | **N.D.** | **N.D.** | **11430.0** | **9427.5** | **2247.2** | **2370.0** |
|  | **Sample 3** | **17.9** | | **15.2** | **N.D.** | **N.D.** | **10455.0** | **12427.5** | **2246.3** | **2682.2** |
|  | **Sample 4** | **50.9** | | **26.6** | **N.D.** | **N.D.** | **14887.5** | **10927.5** | **2190.0** | **2401.9** |
|  | **Sample 5** | **18.8** | | **19.6** | **N.D.** | **N.D.** | **10950.0** | **13035.0** | **2271.6** | **2313.8** |
|  | **Sample 6** | **20.0** | | **21.0** | **N.D.** | **N.D.** | **12975.0** | **10357.5** | **2089.7** | **2592.2** |
|  | **Sample 7** | **24.4** | | **46.9*** | **N.D.** | **N.D.** | **12412.5** | **13612.5** | **2601.6** | **2391.6** |
|  | **Sample 8** | **N.A.** | | **19.8** | **N.A.** | **N.D.** | **N.A.** | **12757.5** | **N.A.** | **2484.4** |
|  | **Sample 9** | **N.A.** | | **28.2** | **N.A.** | **N.D.** | **N.A.** | **12007.5** | **N.A.** | **2473.1** |
|  | **Mean (SEM)** | **25.6 (5.0)** | | **22.7 (1.8)** | **N.D.** | **N.D.** | **12305.4 (570.6)** | **11842.5 (451.4)** | **2320.2 (75.3)** | **2425.9 (53.7)** |
|  | **N** | **7** | | **8** | **N.D.** | **N.D.** | **7** | **9** | **7** | **9** |
|  | **P-value** | **0.59** | | | **N.D.** | | **0.54** | | **0.28** | |

| **Tissue of interest** | | **Insulin (pg/ml)** | | **Igf1 (pg/ml)** | | **Kcnq1 (pg/ml)** | | **Bdnf (pg/ml)** | |
| --- | --- | --- | --- | --- | --- | --- | --- | --- | --- |
|  |  | **TH** | **SWR/J** | **TH** | **SWR/J** | **TH** | **SWR/J** | **TH** | **SWR/J** |
| **Cerebellum** | **Sample 1** | 24.0 | 31.4 | 123.0 | 188.6 | 9525.0 | 11062.5 | 3574.7 | 3804.4 |
|  | **Sample 2** | 60.2 | 36.1 | 109.5 | 224.3 | 13620.0 | 10785.0 | 3106.9 | 3851.3 |
|  | **Sample 3** | 31.9 | 28.8 | 87.2 | 428.0* | 13905.0 | 13110.0 | 4125.9 | 4395.0 |
|  | **Sample 4** | 22.6 | 43.0 | 34.7 | 119.7 | 12795.0 | 11662.5 | 3892.5 | 3712.5 |
|  | **Sample 5** | 82.7 | 31.2 | 92.1 | 177.2 | 8617.5 | 9810.0 | 3967.5 | 4006.9 |
|  | **Sample 6** | 32.2 | 33.0 | 94.8 | 123.5 | 10897.5 | 10710.0 | 4238.4 | 4189.7 |
|  | **Sample 7** | 60.6 | 45.0 | 233.5* | 228.1 | 12562.5 | 12577.5 | 4303.1 | 3807.2 |
|  | **Sample 8** | N.A. | 23.7 | N.A. | 124.1 | N.A. | 11332.5 | N.A. | 3521.3 |
|  | **Sample 9** | N.A. | 28.9 | N.A. | 164.2 | N.A. | 10822.5 | N.A. | 4148.4 |
|  | **Mean (SEM)** | **44.9 (8.7)** | **33.4 (2.3)** | **90.2 (12.3)** | **168.7 (15.5)** | **11703.2 (777.2)** | **11319.2 (336.5)** | **3887.0 (159.3)** | **3937.4 (90.4)** |
|  | **N** | **7** | **9** | **6** | **8** | **7** | **9** | **7** | **9** |
|  | **P-value** | **0.25** | | **0.002** | | **0.66** | | **0.79** | |
| **Blood plasma** | **Sample 1** | N.A. | N.D. | N.A. | 113.8 | N.A. | 88250.0 | N.A. | N.D. |
|  | **Sample 2** | N.D. | N.D. | 2936.4 | 151.7 | 95680.0 | 100330.0 | N.D. | N.D. |
|  | **Sample 3** | N.D. | N.D. | 2581.3 | 50.9 | 97680.0 | 82330.0 | N.D. | N.D. |
|  | **Sample 4** | N.D. | N.D. | 753.1 | 3115.2* | 97500.0 | 96230.0 | N.D. | N.D. |
|  | **Sample 5** | N.D. | N.D. | 563.8 | N.D. | 88180.0 | 54480.0 | N.D. | N.D. |
|  | **Sample 6** | N.D. | N.D. | N.D. | N.D. | 79250.0 | 86700.0 | N.D. | N.D. |
|  | **Sample 7** | N.D. | N.D. | 1687.5 | N.D. | 91480.0 | 87080.0 | N.D. | N.D. |
|  | **Sample 8** | N.A. | N.D. | N.A. | N.D. | N.A. | 76480.0 | N.A. | N.D. |
|  | **Sample 9** | N.A. | N.D. | N.A. | 74.2 | N.A. | 61030.0 | N.A. | N.D. |
|  | **Mean (SEM)** | **N.D.** | **N.D.** | **1217.4 (453.6)** | **97.6 (22.2)** | **91628.3 (2902.0)** | **81434.4 (5069.3)** | **N.D.** | **N.D.** |
|  | **N** | **N.D.** | **N.D.** | **7** | **4** | **6** | **9** | **N.D.** | **N.D.** |
|  | **P-value** | **N.D.** | | **0.049** | | **0.11** | | **N.D.** | |
