# Supplementary material for: Converging evidence points towards a role of insulin signaling in regulating compulsive behavior
Source: Transl Psychiatry. 2019 Sep 12;9:225. doi: 10.1038/s41398-019-0559-6 (PMC6742634; doi:10.1038/s41398-019-0559-6)
Supplement: Supplementary file 7 — Supplementary Table 2 [file 41398_2019_559_MOESM7_ESM.docx]

**Supplementary Table 2: MRS data and correlations with spontaneous alternation behavior**

| **Supplementary Table 2:** The expression levels of nine metabolites obtained via magnetic resonance spectroscopy (MRS) were assessed in the dorsomedial striatrum (DMS) and anterior cingulate cortex (ACC) of TALLYHO/JngJ (TH) and SWR/J mice (n=9 per strain). The metabolites of interest are glutamate (Glu), glutamine (Gln), taurine (Tau), N-acetylaspartate (NAA), sum of myo-Inositol and glycine (mI+Gly), total choline (tCho), glucose (Glc), GABA and glutathione (GSH). MRS data is expressed as ratios of the metabolites relative to the total creatinine (tCr) levels. Only signals with a Cramer-Rao lower bound (CRLB)≤20% were included in the quantification, sometimes resulting in a reduction of the number of animals included in the analyses. The expression data was analyzed using individual T-tests, and correlations with spontaneous alternation behavior were assessed by Pearson correlation. Both datasets were corrected for multiple testing using the False Discovery Rate (FDR) method. The expression data is displayed as mean (SEM). | | | | | | | | |  |
| --- | --- | --- | --- | --- | --- | --- | --- | --- | --- |
| **Region of interest** | **Metabolite of interest** | **TH** | | **SWR/J** | | **T-test** | **Correlation with spontaneous alternation** | |  |
|  |  | **Concentration/tCr** | **N** | **Concentration/tCr** | **N** | **FDR corrected p-value** | **Pearson’s r** | **FDR corrected p-value** | |
| **Dorsomedial striatum** | Glu | 1.160778 (0.038546) | 9 | 1.260875 (0.035288) | 8 | 0.131934566 | 0.081 | 0.757 | |
|  | Gln | 0.710111 (0.032219) | 9 | 0.638625 (0.047421) | 8 | 0.275596649 | -0.234 | 0.366 | |
|  | Tau | 1.553000 (0.041447) | 9 | 1.548625 (0.044132) | 8 | 0.943 | -0.157 | 0.547 | |
|  | NAA | 0.8653 (0.03065) | 9 | 0.8414 (0.02844) | 8 | 0.575 | -0.214 | 0.41 | |
|  | mI+Gly | 0.4854 (0.0253) | 9 | 0.4639 (0.03432) | 8 | 0.621 | -0.059 | 0.822 | |
|  | tCho | 0.1612 (0.00959) | 9 | 0.1621 (0.00743) | 8 | 0.942 | 0.037 | 0.889 | |
|  | Glc | 0.527 (0.02475) | 9 | 0.3079 (0.03489) | 7 | 0.003134765 | -0.658 | 0.006 | |
|  | GABA | 0.254 (0.02913) | 6 | 0.2072 (0.02331) | 5 | 0.25542532 | -0.105 | 0.759 | |
|  | GSH | 0.1594 (0.01079) | 9 | 0.165 (0.02571) | 7 | 0.847 | -0.07 | 0.797 | |
| **Anterior cingulate cortex** | Glu | 1.6277 (0.06447) | 9 | 1.3386 (0.18398) | 9 | 0.222969417 | -0.214 | 0.41 | |
|  | Gln | 0.6937 (0.06322) | 9 | 0.7507 (0.05617) | 8 | 0.51 | -0.267 | 0.3 | |
|  | Tau | 1.1134 (0.06884) | 9 | 0.7725 (0.10376) | 8 | 0.063328592 | -0.352 | 0.166 | |
|  | NAA | 1.0674 (0.08956) | 9 | 0.9535 (0.07189) | 8 | 0.337 | -0.139 | 0.594 | |
|  | mI+Gly | 0.6106 (0.0434) | 9 | 0.466 (0.06364) | 8 | 0.126657183 | -0.276 | 0.283 | |
|  | tCho | 0.444 (0.04553) | 9 | 0.3239 (0.03832) | 8 | 0.13087909 | -0.458 | 0.064 | |
|  | Glc | 0.5724 (0.07957) | 9 | 0.3356 (0.0706) | 7 | 0.113463727 | -0.188 | 0.486 | |
|  | GABA | 0.2538 (0.05349) | 6 | 0.2133 (0.00088) | 3 | 0.424829303 | -0.149 | 0.702 | |
|  | GSH | 0.3254 (0.02139) | 9 | 0.2118 (0.02792) | 6 | 0.047496444 | -0.222 | 0.427 | |
