# Supplementary material for: Converging evidence points towards a role of insulin signaling in regulating compulsive behavior
Source: Transl Psychiatry. 2019 Sep 12;9:225. doi: 10.1038/s41398-019-0559-6 (PMC6742634; doi:10.1038/s41398-019-0559-6)
Supplement: Supplementary file 8 — Supplementary Table 3 [file 41398_2019_559_MOESM8_ESM.docx]

**Supplementary Table 3: DTI data and correlations with spontaneous alternation behaviour**

| **Supplementary Table 3:** Fractional anisotropy (FA) and mean diffusivity (MD) was assessed in six brain regions in TALLYHO/JngJ (TH) and SWR/J mice (n=9 per strain). The brain regions of interest are the anterior cingulate cortex (ACC), dorsomedial striatum (DMS), corpus callosum (CC), superior cerebellar peduncle (SCP), orbitofrontal cortex (OFC) and nucleus accumbens (NAcc). The data was analyzed using individual T-tests, and correlations with spontaneous alternation behavior were assessed by Pearson correlation. Both datasets were corrected for multiple testing using the False Discovery Rate (FDR) method. The expression data is displayed as mean (SEM). | | | | | | | |
| --- | --- | --- | --- | --- | --- | --- | --- |
| **Fractional anisotropy (FA)** | | | | | | | |
| **Region of interest** | **TH** | | **SWR/J** | | **T-test** | **Correlation with signal attenuation** | |
|  | **Mean (SEM)** | **N** | **Mean (SEM)** | **N** | **FDR corrected p-value** | **Pearson’s r** | **FDR corrected p-value** |
| ACC | 0.205056 (0.007075) | 9 | 0.203056 (0.005683) | 9 | 0.83 | -0.154 | 0.54 |
| DMS | 0.175275 (0.007285) | 9 | 0.201961 (0.005615) | 9 | 0.01 | 0.734 | 0.002 |
| CC | 0.547256 (0.008226) | 9 | 0.478372 (0.009885) | 9 | 0.000037 | -0.336 | 0.17 |
| SCP | 0.280056 (0.019628) | 9 | 0.362383 (0.012722) | 9 | 0.003 | 0.548 | 0.02 |
| OFC | 0.189142 (0.013421) | 9 | 0.186044 (0.013558) | 9 | 0.87 | 0.027 | 0.91 |
| NAcc | 0.149189 (0.007170) | 9 | 0.163744 (0.016914) | 9 | 0.45 | 0.211 | 0.40 |
| **Mean diffusivity (MD)** | | | | | | | |
| **Region of interest** | **TH** | | **SWR/J** | | **T-test** | **Correlation with signal attenuation** | |
|  | **Mean (SEM)** | **N** | **Mean (SEM)** | **N** | **FDR corrected p-value** | **Pearson’s r** | **FDR corrected p-value** |
| ACC | 0.000756 (0.000010) | 9 | 0.000762 (0.000007) | 9 | 0.63 | -0.245 | 0.33 |
| DMS | 0.000717 (0.000011) | 9 | 0.000758 (0.000012) | 9 | 0.02 | 0.275 | 0.27 |
| CC | 0.000744 (0.000006) | 9 | 0.000778 (0.000012) | 9 | 0.03 | 0.393 | 0.11 |
| SCP | 0.000650 (0.000029) | 9 | 0.000744 (0.000013) | 9 | 0.01 | 0.42 | 0.08 |
| OFC | 0.000753 (0.000018) | 9 | 0.000772 (0.000020) | 9 | 0.48 | -0.033 | 0.90 |
| NAcc | 0.000694 (0.000006) | 9 | 0.000728 (0.000022) | 9 | 0.18 | 0.147 | 0.56 |
