# Supplementary material for: Converging evidence points towards a role of insulin signaling in regulating compulsive behavior
Source: Transl Psychiatry. 2019 Sep 12;9:225. doi: 10.1038/s41398-019-0559-6 (PMC6742634; doi:10.1038/s41398-019-0559-6)
Supplement: Supplementary file 9 — Supplementary Table 4 [file 41398_2019_559_MOESM9_ESM.docx]

**Supplementary Table 4: Correlation analyses between MRS and DTI data sets**

| **Supplementary Table 4:** Correlation analyses between magnetic resonance spectroscopy (MRS) and diffusor tensor imaging (DTI) data sets. The expression levels of nine metabolites obtained via magnetic resonance spectroscopy (MRS) were assessed in the dorsomedial striatrum (DMS) and anterior cingulate cortex (ACC) of TALLYHO/JngJ (TH) and SWR/J mice (n=9 per strain). In the same brain regions, the Fractional anisotropy (FA) and mean diffusivity (MD) were assessed. The metabolites of interest are glutamate (Glu), glutamine (Gln), taurine (Tau), N-acetylaspartate (NAA), sum of myo-Inositol and glycine (mI+Gly), total choline (tCho), glucose (Glc), GABA and glutathione (GSH). Correlations between the DTI markers and metabolites were assessed by Pearson correlation, and corrected for multiple testing using the False Discovery Rate (FDR) method. | | | | | |
| --- | --- | --- | --- | --- | --- |
| **Brain region** | **DTI marker** | **Metabolite** | **Pearson correlation** | | |
|  |  |  | **Pearson’s r** | **FDR corrected p-value** | **N** |
| **DMS** | **FA** | Glu | 0.246 | 0.331 | 17 |
|  |  | Gln | -0.265 | 0.334 | 17 |
|  |  | Tau | -0.184 | 0.377 | 17 |
|  |  | NAA | -0.33 | 0.323 | 17 |
|  |  | mI + Gly | -0.204 | 0.377 | 17 |
|  |  | tCho | -0.185 | 0.394 | 17 |
|  |  | Glc | -0.576 | 0.314 | 16 |
|  |  | GABA | -0.183 | 0.348 | 11 |
|  |  | GSH | -0.181 | 0.362 | 16 |
|  | **MD** | Glu | -0.01 | 0.458 | 17 |
|  |  | Gln | -0.397 | 0.238 | 17 |
|  |  | Tau | -0.127 | 0.358 | 17 |
|  |  | NAA | 0.002 | 0.456 | 17 |
|  |  | mI + Gly | 0.183 | 0.362 | 17 |
|  |  | tCho | -0.068 | 0.412 | 17 |
|  |  | Glc | -0.444 | 0.234 | 16 |
|  |  | GABA | 0.204 | 0.362 | 11 |
|  |  | GSH | 0.104 | 0.375 | 16 |
| **ACC** | **FA** | Glu | -0.494 | 0.153 | 18 |
|  |  | Gln | 0.046 | 0.419 | 17 |
|  |  | Tau | 0.141 | 0.360 | 17 |
|  |  | NAA | -0.349 | 0.311 | 17 |
|  |  | mI + Gly | 0.162 | 0.367 | 17 |
|  |  | tCho | -0.147 | 0.364 | 17 |
|  |  | Glc | -0.256 | 0.349 | 16 |
|  |  | GABA | -0.395 | 0.346 | 9 |
|  |  | GSH | -0.061 | 0.415 | 15 |
|  | **MD** | Glu | 0.119 | 0.351 | 18 |
|  |  | Gln | 0.535 | 0.149 | 17 |
|  |  | Tau | -0.543 | 0.198 | 17 |
|  |  | NAA | 0.229 | 0.346 | 17 |
|  |  | mI + Gly | -0.308 | 0.344 | 17 |
|  |  | tCho | -0.29 | 0.357 | 17 |
|  |  | Glc | -0.511 | 0.142 | 16 |
|  |  | GABA | 0.412 | 0.343 | 9 |
|  |  | GSH | -0.437 | 0.246 | 15 |
