# Supplementary material for: Converging evidence points towards a role of insulin signaling in regulating compulsive behavior
Source: Transl Psychiatry. 2019 Sep 12;9:225. doi: 10.1038/s41398-019-0559-6 (PMC6742634; doi:10.1038/s41398-019-0559-6)
Supplement: Supplementary file 10 — Supplementary Table 5 [file 41398_2019_559_MOESM10_ESM.docx]

**Supplementary Table 5: Correlations between proteomics data and signal attenuation behavior.**

| **Supplementary Table 5:** Protein expression levels of insulin, Igf1, Kcnq1 and Bdnf were assessed in the prefrontal cortex, striatum, cerebellum and blood plasma of TALLYHO/JngJ (TH) and SWR/J mice. Data are displayed as mean (SEM). N.D. indicates that the analysis could not be performed for this type of tissue or protein due to limitations in the availability of the tissue. Correlations with spontaneous alternation behavior were assessed by Pearson correlation, and corrected for multiple testing using the False Discovery Rate (FDR) method. | | | |
| --- | --- | --- | --- |
| **Tissue of interest** | **Protein of interest** | **Correlation with signal attenuation** | |
|  |  | **Pearson’s r** | **FDR corrected p-value** |
| **Prefrontal cortex** | **Insulin** | 0,039 | 0,89 |
|  | **Igf1** | 0,004 | 0,99 |
|  | **Kcnq1** | -0,063 | 0,85 |
|  | **Bdnf** | 0,335 | 0,32 |
| **Striatum** | **Insulin** | 0,359 | 0,44 |
|  | **Igf1** | N.D. | N.D. |
|  | **Kcnq1** | 0,275 | 0,40 |
|  | **Bdnf** | 0,341 | 0,37 |
| **Cerebellum** | **Insulin** | -0,416 | 0,34 |
|  | **Igf1** | 0,48 | 0,77 |
|  | **Kcnq1** | -0,008 | 0,98 |
|  | **Bdnf** | 0,012 | 0,97 |
| **Blood plasma** | **Insulin** | N.D. | N.D. |
|  | **Igf1** | -0,517 | 0,48 |
|  | **Kcnq1** | -0,18 | 0,61 |
|  | **Bdnf** | N.D. | N.D. |
